# Biomodulatory Treatment of Patients with Castration-Resistant Prostate Cancer: A Phase II Study of Imatinib with Pioglitazone, Etoricoxib, Dexamethasone and Low-Dose Treosulfan

**DOI:** 10.1007/s12307-014-0161-7

**Published:** 2014-12-11

**Authors:** M. Vogelhuber, S. Feyerabend, A. Stenzl, T. Suedhoff, M. Schulze, J. Huebner, R. Oberneder, W. Wieland, S. Mueller, F. Eichhorn, H. Heinzer, K. Schmidt, M. Baier, A. Ruebel, K. Birkholz, A. Bakhshandeh-Bath, R. Andreesen, W. Herr, A. Reichle

**Affiliations:** 1 Department of Hematology and Oncology, University Hospital Regensburg, Franz-Josef-Strauß-Allee 11, 93053 Regensburg, Germany; 2 Department of Urology, University Hospital Tuebingen, Hoppe-Seyler-Strasse 3, 72076 Tuebingen, Germany; 3 Department of Hematology and Oncology, Hospital Passau, Innstrasse 76, 94032 Passau, Germany; 4 Outpatient Center for Urology and Oncology, Hauptstrasse 10, 04416 Markkleeberg, Germany; 5 Department of Oncology, J. W. Goethe University, Theodor-Stern-Kai 7, 60323 Frankfurt, Germany; 6 Urologic Hospital München-Planegg, Germeringer Str. 32, 82152 Planegg, Germany; 7 Department of Urology, Hospital St. Josef, University Regensburg, Landshuter Strasse 65, 93053 Regensburg, Germany; 8 Department of Urology, University Hospital Bonn, Sigmund-Freud-Strasse 25, 53105 Bonn, Germany; 9 Outpatient Center, Rinckstrasse 7-9, 83435 Bad Reichenhall, Germany; 10 Martini-Clinic at University Hospital Hamburg-Eppendorf, Martinistrasse 52, 20246 Hamburg, Germany; 11 Novartis Pharma GmbH, Roonstrasse 25, 90429 Nuernberg, Germany; 12 Outpatient Center for Medical Oncology, Waitzstrasse 22, 22607 Hamburg, Germany

**Keywords:** Castration-resistant prostate cancer, Multi-targeted, biomodulatory therapy, PSA response, Quality of life

## Abstract

Therapeutic options for patients with castration-resistant prostate cancer (CRPC) remain limited. In a multicenter, Phase II study, 65 patients with histologically confirmed CRPC received a biomodulatory regimen during the six-month core study. Treatment comprised daily doses of imatinib mesylate, pioglitazone, etoricoxib, treosulfan and dexamethasone. The primary endpoint was prostate-specific antigen (PSA) response. Responders could enter an extension phase until disease progression or intolerable toxicity occurred. Mean PSA was 45.3 ng/mL at baseline, and 77 % of patients had a PSA doubling time <3 months. Of the 61 evaluable patients, 37 patients (60.6 %) responded or had stable disease and 23 of them (37.7 % of 61 patients) were PSA responders. Among the 23 responders mean PSA decreased from 278.9 ± 784.1 ng/mL at baseline to 8.8 ± 11.6 ng/mL at the final visit (week 24). The progression-free survival (PFS) was 467 days in the ITT population. Of the 947 adverse events, 57.6 % were suspected to be drug-related, 13.8 % led to dose adjustment or permanent discontinuation and 40.2 % required concomitant medication. This novel combination approach led to an impressive PSA response rate of 37.7 % in CRPC patients. The good PSA response and PFS rate combined with the manageable toxicity profile suggest an alternative treatment option.

## Introduction

In recent years, a variety of novel compounds have shown a survival benefit in castration-resistant prostate cancer (CRPC) including the novel taxane cabazitaxel, sipuleucel-T, radium-223 chloride (alpharadin), the androgen biosynthesis inhibitor abiraterone acetate, and the androgen receptor inhibitor enzalutamide [1–3]. Further androgen receptor and androgen biosynthesis inhibitors, immune-modulating compounds (PROSTVAC-VF), as well as angiogenesis inhibitors (thalidomide, lenalidomine, tasquinimod) and orteronel (TAK-700) are currently being investigated [2]. The availability of these new therapeutic approaches has made it possible to explore the use of sequential treatment regimens in an attempt to balance the risks and benefits of novel compounds for individual CRPC patients.

We report here the findings of a Phase II, single-arm, multicenter trial in which CRPC patients received a combination of agents with concerted biomodulatory activity, but poor monoactivity. This combined biomodulatory approach aims at targeting rationalizations of particular hallmarks that means the physical organization of cancer [4], such as tumor-associated inflammation, angiogenesis and immune response. Each of these is highly relevant in prostate cancer: inflammation plays a crucial role in its pathogenesis [5], tumor-associated angiogenesis is obligate, and prostate cancer is known to be a principally immunogenic tumor [6]. In addition, the current regimen targets the contribution made by osteoblasts to tumor growth in CRPC since osteoblastic metastases make up up to 80 % of the organ metastases in prostate cancer [7]. The aim of the multi-targeted treatment approach is to modulate and redirect the function of tumor and stroma cells via ubiquitous, non-oncogene addicted targets, similar to a recently published trial [8]. The present trial combines the PDGFR inhibitor (imatinib) [9, 10] as antiangiogenic [11] component with the PPARalpha/gamma receptor agonist (pioglitazone) [12–15] and the glucocorticoid receptor agonist (dexamethasone) [16–19] as epigenetically modeling drugs with anti-inflammatory and anti-osteoblastic activity, and the cyclooxygenase-2 inhibitor (etoricoxib) [20] as anti-inflammatory component. Such biomodulatory effects are coupled with the pleiotropic immunomodulatory and angiostatic activity of metronomic low-dose chemotherapy using treosulfan, via regulatory T-cells and thrombospondin-1, respectively [21]. Two of the study drugs, dexamethasone [16–19] and metronomic low-dose chemotherapy with alkylating agents [21–24] have previously shown monoactivity in CRPC. Others have demonstrated activity in in vitro or animal models, but failed to induce a response in vivo (pioglitazone [13]) or to improve the response when added to taxotere (imatinib [10]).

## Patients and Methods

### Study Design and Conduct

**Fig. 1:**
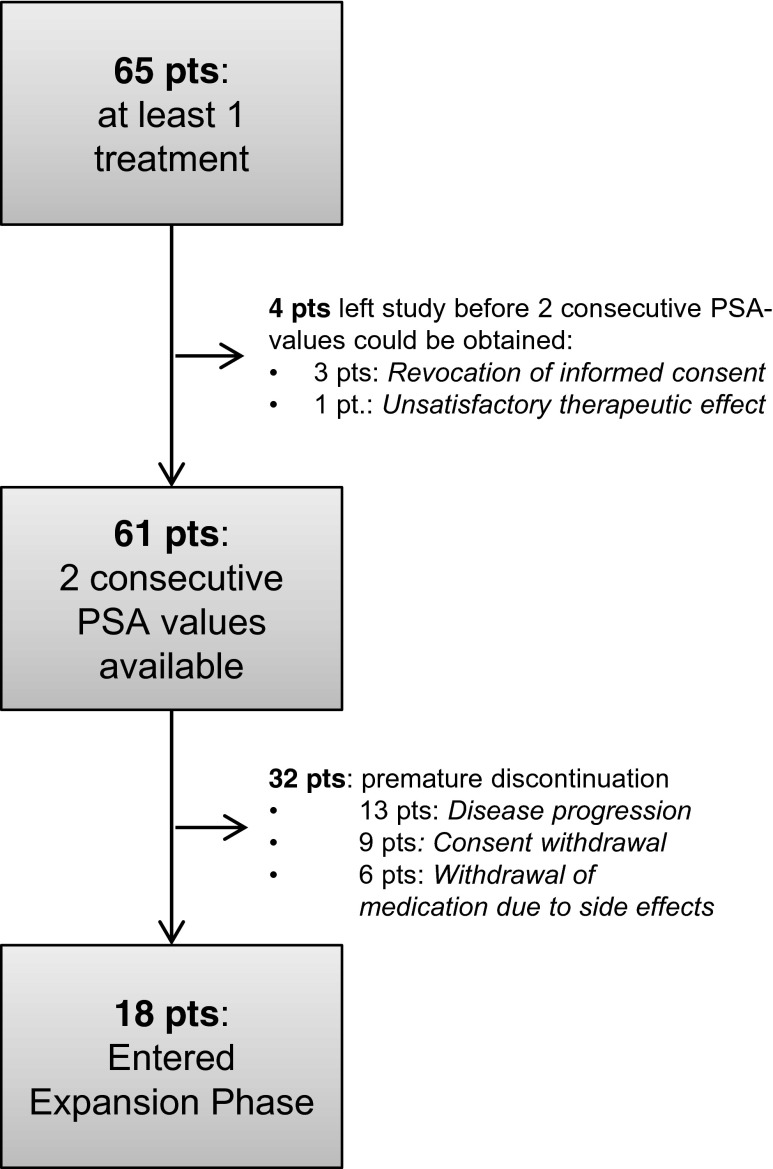
Study design including the main inclusion and exclusion criteria. *ECOG* Eastern cooperative oncology group performance status, *CRPC* Castration-resistant prostate cancer, *PSA* Prostate-specific antigen, *AE* Adverse event, *SAE* Serious adverse event

This was a single-arm, open-label, six-month Phase II study undertaken at 11 German centers in which CRPC patients received imatinib mesylate, pioglitazone, etoricoxib, treosulfan and dexamethasone until progression of prostate-specific antigen (PSA) (Fig. 1). For the drug combination, no data about potential drug-drug interactions concerning side effects are available. At the end of the core six-month study, patients with a PSA decrease ≥30 % from baseline and 24 weeks of treatment without any signs of disease progression were followed until disease progression or intolerable toxicity occurred. The study protocol was approved by the institutional review board of the participating centers and by the health authorities and written informed consent from patients was obtained before enrollment.

The trial was sponsored by Novartis Pharma GmbH and registered at ClinicalTrials.gov (NCT: NCT00427999).

### Study Population

Male patients ≥18 years who had histologically confirmed prostate cancer with proven progression after androgen deprivation therapy (surgical or medical castration) were eligible. Patients were required to have total serum testosterone <1.72 nmol/L (50 ng/dL) and to be castration-resistant, defined as three consecutive elevated (≥50 % above nadir) serum PSA tests separated by at least 2 weeks, with the last two PSA measurements ≥5.0 ng/mL despite secondary hormonal manipulations (according to European Association of Urology (EAU) guidelines) [25]. Additional inclusion criteria were Eastern Cooperative Oncology Group (ECOG) performance status ≤2, adequate hematological status (defined as absolute neutrophil count >1,500/mm^3^, platelet count >75,000/mm^3^), normal hepatic, renal and cardiac function, and a life expectancy of at least 6 months.

Key exclusion criteria included prior use of chemotherapy, prior treatment with imatinib or other tyrosine kinase inhibitors, concomitant therapy with other tumor treatments except for LHRH agonists, concomitant warfarin, phenprocoumon or other oral anticoagulant treatment, radiotherapy of >25 % of the bone marrow, systemic radioisotope therapy, uncontrolled brain metastases, regular blood transfusions, and previous secondary malignant disease within the past 5 years. Patients with the following comorbidities were excluded: symptomatic congestive heart failure, unstable angina or recent myocardial infarction, uncontrolled diabetes, chronic hepatic or renal disease, active uncontrolled infection, chronic inflammatory intestinal disease, autoimmune disease or a known diagnosis of HIV, hepatitis B or C infection.

### Intervention

Eligible patients received oral daily doses of imatinib mesylate (400 mg daily), pioglitazone (60 mg daily), etoricoxib (60 mg daily), treosulfan (250 mg twice daily) and dexamethasone (1 mg daily) until PSA progression. Patients with PSA progression were switched to a dose of 400 mg imatinib twice daily and at the same time treosulfan was reduced to 250 mg daily; if further progression occurred, patients were withdrawn from the study. Dose reductions were permitted for intolerable non-hematologic or hematologic grade 2 toxicity or any grade 3 or 4 toxicity. Depending on the kind of toxicity, the dose of one of the study drugs was reduced at the investigator’s discretion. The minimum required medication was a combination of treosulfan 250 mg daily and one biomodulator (etoricoxib or pioglitazone or imatinib mesylate) and dexamethasone following a 4 week interruption due to Grade 2/3/4 toxicity. Study medication was restarted after the toxicity of the respective drug(s) had resolved or improved to less than grade 2 or less than grade 3, depending on the toxicity and respective drug. If toxicity recurred despite dose reduction(s), the relevant drug was withdrawn. Dose reductions, if required, were specified as follows: reduced dose for imatinib depending on dose and toxicity grade (between 200 and 600 mg/day), 60 mg every second day for etoricoxib, 45 mg/day for pioglitazone, 0.5 mg/day for dexamethasone and 250 mg/day for treosulfan. Study withdrawal was to take place, if medication could not be maintained at a minimum of treosulfan 250 mg/day plus one biomodulator (etoricoxib, or pioglitazone or imatinib) plus dexamethasone following a four-week interruption due to grade 2–4 toxicity. Patients were also to discontinue the study if they refused to continue therapy, or in response to protocol violations or administrative problems. Concomitant use of bisphosphonates was allowed.

### Evaluation

During the screening all patients underwent imaging by CT, MRI, or plain radiography as necessary to confirm metastatic sites (only assessed if performed in clinical routine). A radioisotope bone scan was performed in patients with bone metastases. Pre-treatment evaluations included medical history, ECOG performance status, vital signs, physical examination, electrocardiogram, laboratory screening including PSA and testosterone levels, coagulation assessment, urinalysis, electrocardiography and assessment of quality of life (EORTC-30 questionnaire [26]). During the six-month core study, PSA values, ECOG performance status and quality of life were assessed monthly. Physical examination, vital signs and blood tests were performed after 1, 2, 4, 8, 12 and 16 weeks, coagulation was measured after 4 weeks, and subsequently if clinically indicated. Urinalysis and imaging by CT, MRI, plain radiography or bone scanning were performed as clinically indicated. At the final visit of the core study, ECOG performance status, vital signs and concomitant medication/therapies were recorded, and physical examination, laboratory screening including PSA, coagulation and urinalysis, quality of life assessment and imaging (if clinically indicated) were performed. Adverse events were monitored throughout the study and were graded according to the National Cancer Institute Common Terminology Criteria for adverse events (version 3.0).

### Study Endpoints

The primary endpoint was PSA response, defined as a reduction in serum PSA ≥50 % in patients compared to baseline value that was confirmed by a second PSA value 3–4 weeks later [25]. According to Kelly et al., assessment of PSA response is a reliable tool to analyze therapy success and is in line with the results of radiologic assessment [27]. Patients not fulfilling these criteria were defined as PSA non-responders and were categorized as having PSA progression or stable disease. PSA progression was defined as a PSA increase of at least 50 % over baseline or an increase of at least 25 % over baseline with an absolute PSA increase of 5 ng/L, which had to be confirmed 3 to 4 weeks later. PSA non-responders were considered to have stable disease, if they did not meet the criteria for progressive disease [25].

Secondary endpoints included the time to PSA response (defined as the time from first administration of study drugs to the first confirmed PSA response), progression-free survival (defined as the time from first administration of study drugs to the first date of PSA progression), overall survival during the extension phase of the study, quality of life including pain response, and safety and tolerability of the combined therapy.

### Statistical Analysis

The sample size calculation estimated that 46 evaluable patients would be required to distinguish between the two rates 40 % (*p*_*1*_) and 25 % (*p*_*0*_) with a one-sided alpha of 10 % and 80 % power, and assuming a 20 % dropout rate [28]. The estimate of a 25 % PSA response rate to glucocorticoid therapy is also retrospectively justified by the 24 % PSA response rate for prednisone in a randomized phase III trial for asymptomatic or weakly symptomatic patients with CRPC [1]. Sample size was estimated based on exact binomial probabilities. The first design (and hence the lowest number) which satisfied the design criteria (alpha and power constraints) was chosen. Assuming a 20 % dropout rate, 58 patients had thus to be included in the trial to achieve 46 evaluable patients.

The intent-to-treat (ITT) population was defined as patients who received at least one dose of all medications and had two available consecutive post-baseline PSA values. The number of PSA responders is presented with the corresponding 90 % confidence interval for ITT population. The median times for PSA response and PFS were calculated for the ITT population. Non-responders were censored with the date of final visit or premature discontinuation. Data on quality of life are presented descriptively.

Progression free survival as well as overall survival were estimated with the Kaplan-Meier method.

## Results

### Patients

**Fig. 2:**
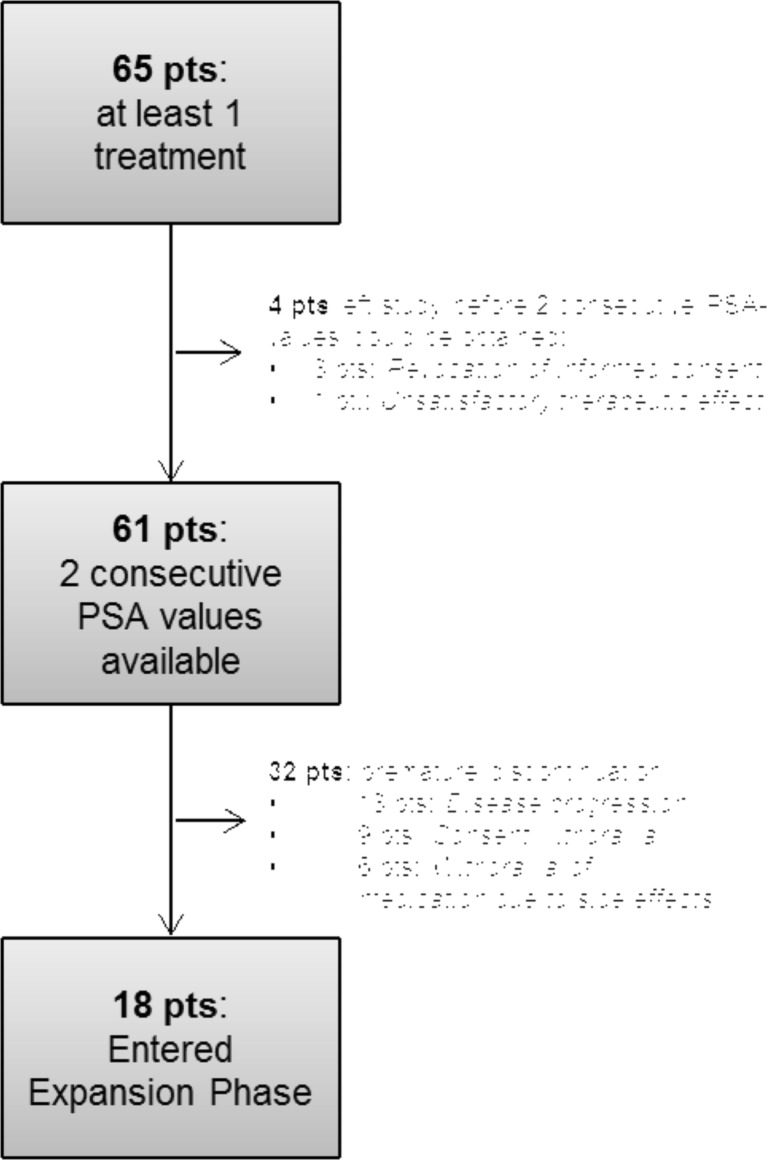
Proportion of patients discontinuing study drug. 1 patient is still ongoing (at the time of manuscript submission, Oct 2014). *Pts* Patients, *PSA* Prostate-specific antigen

Between February 2007 and October 2009, 65 patients received at least one treatment with study medication. As shown in Fig. 2, 61 patients provided two consecutive PSA values and could therefore be included in the ITT population, whereas 4 patients left the study before two consecutive PSA values were measured (rejection of informed consent (3 pts), unsatisfactory therapeutic effect (1 pt)). Thirty-two discontinued the study prematurely, most frequently due to disease progression (*n* = 13), consent withdrawal (*n* = 9) or withdrawal of medication due to side effects (*n* = 6). The mean time on study in the core phase was 141 days, and the mean duration of at least minimal therapy was 121 days. Eighteen patients entered the extension phase, which is still ongoing. One patient has been followed since June 2008 without disease progression or intolerable toxicity. Mean PSA at baseline was 45.3 ng/mL, with values ranging from 5 to 3603 ng/mL. Approximately 78 % of patients had bone metastasis, 34 % had measurable lymph node involvement, and 8 % had measurable organ involvement (Table 1). In total, 77 % of patients had a PSA doubling time of less than 3 months.

**Table 1:** Patient demographics and clinical characteristics (ITT population, *N* = 61)

Age, mean (range)	67 (50–83)
PSA at baseline, mean (range) (ng/mL)	45.3 (5–3603)
ECOG performance status	
ECOG 0, N (%)	49 (80.3)
ECOG 1, N (%)	11 (18.0)
ECOG 2, N, (%)	1 (1.6)
Previous therapy, N (%)	
Prostatectomy	22 (36.1)
Radiation	35 (57.4)
Hormone therapy	61 (100)
Metastases at baseline, N (%)	
Bone metastases only	33 (54.1)
Bone metastases	
+ lung/liver	2 (3.3)
+ lung	1 (1.6)
+ liver	2 (3.3)
+ nodes	13 (21.3)
Nodes only	6 (9.8)
Locally advanced disease	4 (6.6)

*ITT* Intent-to-treat, *PSA* Prostate-specific antigen, *ECOG* Eastern cooperative oncology group performance status

### PSA Response

**Fig. 3:**
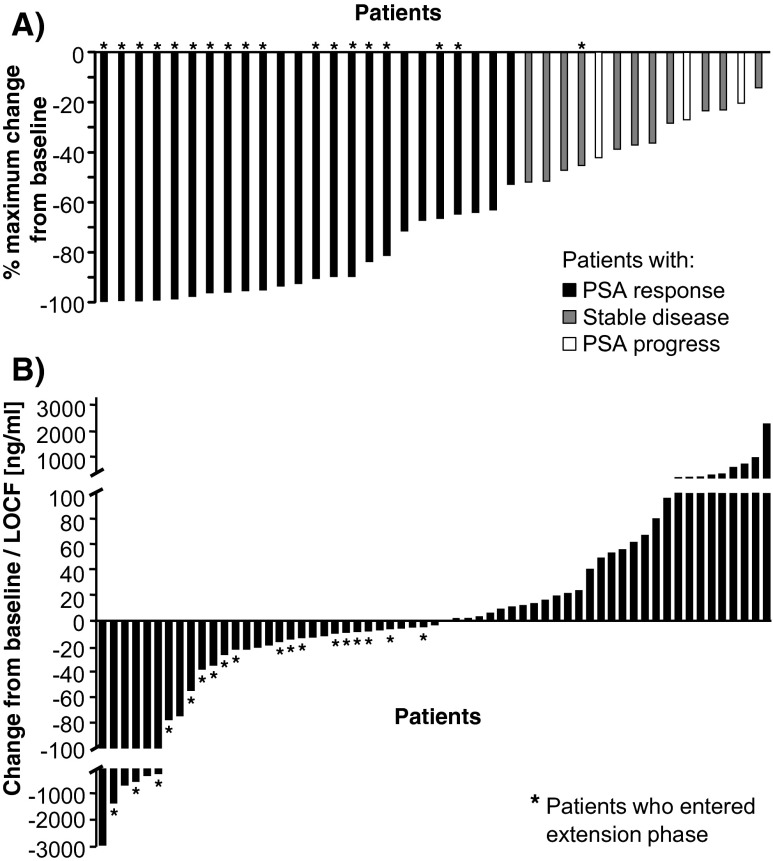
PSA change during therapy. **a** Maximal PSA reduction compared to baseline for patients with PSA response, with stable disease and PSA progress. **b** the PSA change from baseline or LOCF. Patients with PSA response: *black bars*, patients with stable disease: *grey bars*, patients with PSA progress: *white bars. PSA* Prostate-specific antigen, *LOCF* Last observation carried forward, *Patients who entered the expansion phase of the study

PSA response occurred in 23 patients (37.7 %, 90 % CI, 27.5, 47.9). Among responders, PSA decreased in average from 278.9 ± 784.1 ng/mL at baseline to 8.8 ± 11.6 ng/mL at their last visit. The remaining 38 patients (62.3 %, 90 % Cl, 52.1, 72.5) were considered PSA non-responders, of whom 14 patients (14/61, 23.0 %) showed stable disease. Altogether, 37 patients (60.6 %) responded or had stable PSA levels during the study. In total, 26 patients (42.6 %) had a PSA decline ≥50 % and further 12 patients (19.6 %) had PSA reduction <50 % during the six-month core study. PSA reduction >50 % was also observed in five patients with a baseline PSA doubling time <3 months. Figure 3 presents PSA changes by patient from baseline. Twelve patients received an increase in imatinib dose from 400 to 800 mg due to PSA progression, but this did not lead to an improved PSA response. PSA response occurred independent of the presence of distant metastases and the metastatic site. Although 77 % of patients required some type of dose modification or temporary interruption of study drug according to non-hematologic or hematologic toxicities, over 60 % of the population showed either a PSA response or maintained a stable disease outcome.

### Secondary Endpoints

**Fig. 4:**
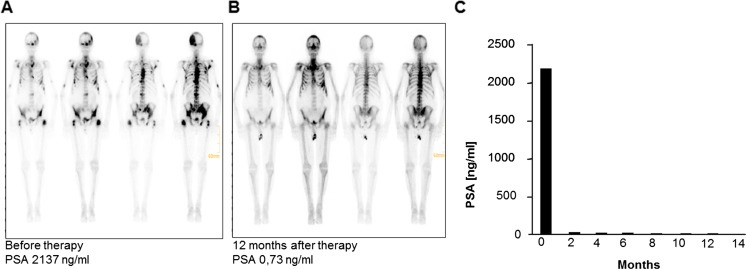
Resolution of bone lesions and evolution of PSA in an 80-year old patient. Bone lesions **a** before and **b** 12 months after therapy. **c** PSA level over time. *PSA* Prostate-specific antigen

**Fig. 5:**
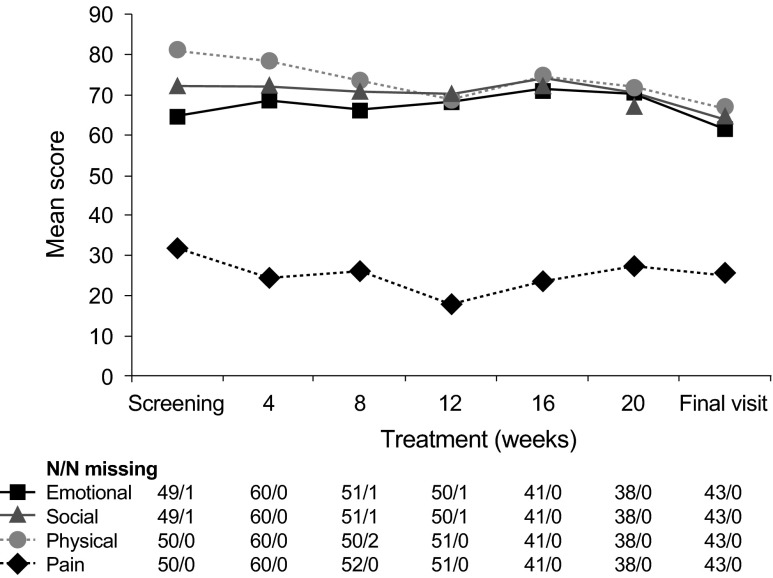
Quality of life: Emotional, social, physical and pain scores per visit. Emotional: *squares*, social: *triangle*, physical: *circle*, pain: *N/N* missing indicates the number of values/number of missing values

**Fig. 6:**
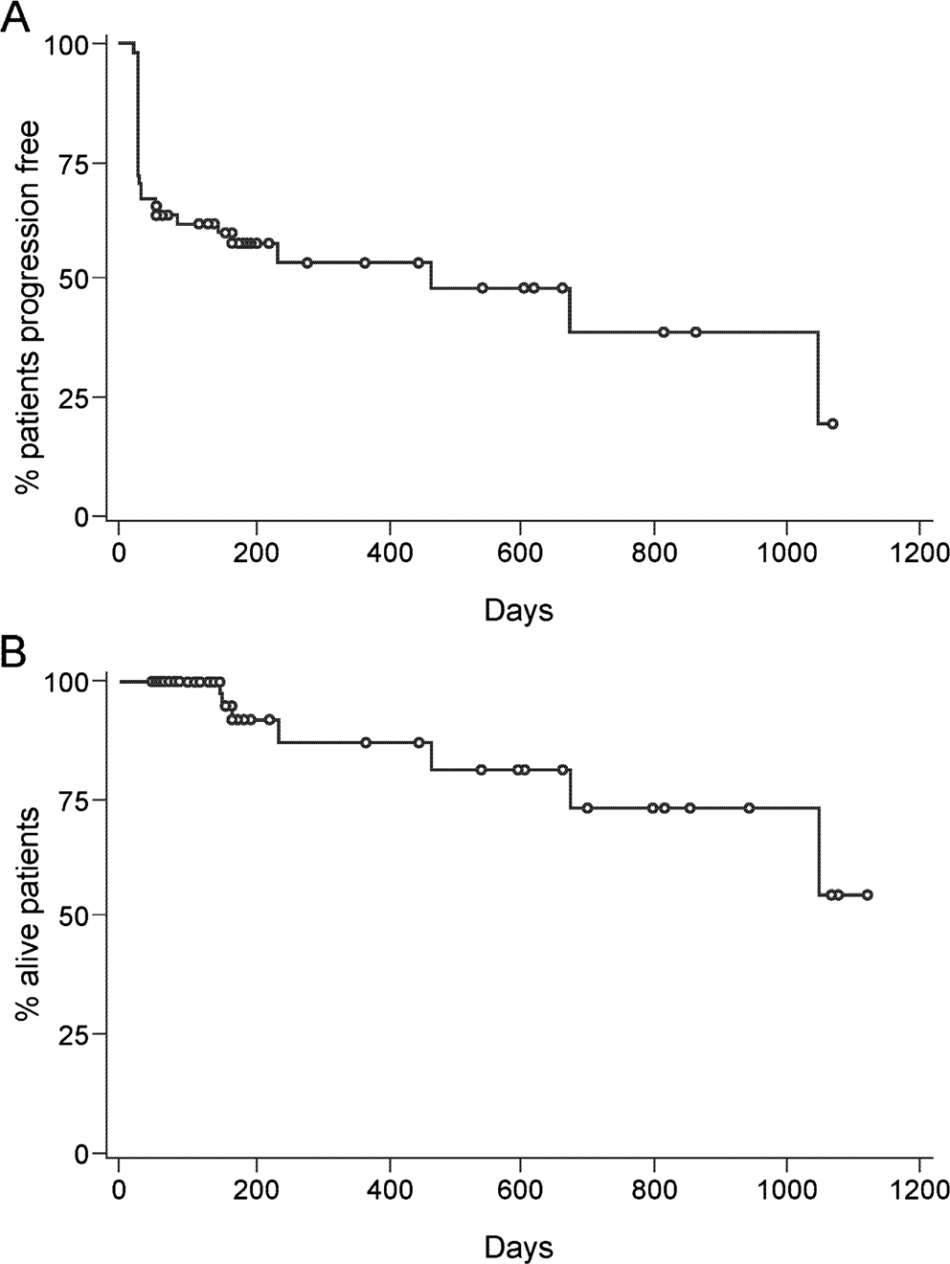
Median progression-free survival (**a**) and overall survival (**b**). *Circle* Censored observation

At one center bone scans were systematically performed on a routine basis. A marked reduction or complete regression of bone lesions could be observed in 6 out of 16 patients (follow-up bone scans did not belong to routine diagnostic investigations according to protocol). Figure 4 shows an example of a patient who experienced a steep decrease of PSA level (from 2137 to 0.73 ng/mL at month 12) accompanied by an impressive improvement in bone lesions. Two patients with extensive lymphatic metastases showed calcifications in lymph node tissue and partial remission according to RECIST criteria. Quality of life assessment showed that social, emotional, pain and physical function scores remained stable throughout the core phase of the study (Fig. 5). Median progression-free survival was 467 days, median overall survival has not been achieved yet (Fig. 6).

### Safety

All 65 patients experienced one or more adverse event (Table 2). The majority of adverse events, 97.1 % (*n* = 920) did not necessitate to permanently discontinue study medication. Of the 947 adverse events, 57.6 % (*n* = 545) were suspected to be drug-related, 13.8 % (*n* = 131) led to dose adjustment or temporary interruption, 2.9 % (*n* = 27) led to permanent discontinuation and 40.2 % (*n* = 381) required concomitant medication or non-drug therapy. The most frequently reported drug-related adverse events were peripheral edema (56.9 %), nausea (38.5 %), edema (36.9 %), fatigue (35.4 %), dyspnea (35.4 %), anemia (33.8 %), leukopenia (29.2 %), diarrhea (23.1 %), vomiting (23.1 %), facial edema (23.1 %), muscle spasms (21.5 %) and increased weight (21.5 %). The most frequently reported adverse events irrespective of relation to any study drug are depicted in Table 2. In total, 98 serious adverse events were reported in 27 patients (41.5 %), of which 32 events in 14 patients were drug-related and led to permanent study drug discontinuation in five cases. The most frequent drug-related serious adverse events were general disorders and administration site conditions (7.7 %), blood and lymphatic system disorders (6.2 %), infections and infestations (4.6 %), nervous system disorders (4.6 %) and gastrointestinal disorders (3.1 %). Four patients (6.2 %) died during the core study as a result of tumor progression (1), acute respiratory insufficiency due to progression of prostate cancer (1), acute respiratory distress syndrome due to pneumonia (1) and cardiac arrest and pulmonary arrest (1) due to extended pulmonary metastatic disease. All deaths were not related to study drugs.

**Table 2:** Adverse events and serious adverse events

	Patients N (%)	AE N (%)
All adverse events	65 (100)	947 (100)
With suspected relation to study drug	64 (98.5)	545 (57.6)
Leading to dose adjustment or temporary interruption	50 (76.9)	131 (13.8)
Leading to permanent discontinuation	15 (23.1)	27 (2.9)
Requiring concomitant medication/non-drug therapy	62 (95.4)	381 (40.2)
All serious adverse events	27 (41.5)	98 (10.3)
Deaths	4 (6.2)	
With suspected relation to study drug	14 (21.5)	32 (3.4)
Leading to permanent discontinuation	5 (7.7)	11 (1.2)
	Patients N (%)	
Frequent adverse events (>20 %)^a^	All grades	Grade ≥3
Peripheral edema	38 (58.5)	1 (1.5)
Nausea	30 (46.2)	3 (4.6)
Fatigue	29 (44,6)	8 (12.3)
Diarrhea	29 (44.6)	0 (0.0)
Dyspnea	26 (40.0)	5 (6.2)
Edema	25 (38.5)	0 (0.0)
Anemia	24 (36.9)	4 (6.2)
Leukopenia	20 (30.8)	5 (7.7)
Vomiting	19 (29.2)	2 (3.1)
Muscle cramps	16 (24.6)	1 (1.5)
Facial edema	15 (23.1)	2 (3.1)
Increased weight	14 (21.5)	0 (0.0)
Increased blood lactate dehydrogenase	14 (21.5)	1 (1.5)

^a^ Irrespective of relation to study drug

## Discussion

Results of this Phase II study suggest that a multi-track therapy approach with the oral biomodulatory active drugs imatinib, treosulfan, etoricoxib, pioglitazone and dexamethasone induces PSA responses of ≥50 % in almost 40 % of patients when used as first-line therapy for mostly rapidly progressing CRPC (77 % <3 months PSA doubling time at base-line). This response rate is comparable with that achieved using standard chemotherapy such as docetaxel (45 %) or mitoxantrone (32 %) [28], and presumably much higher than in ‘low-risk’ patients receiving glucocorticoids only, particularly dexamethasone (up to >30 %) [16–19]. Phase II trials using abiraterone achieved PSA response rates between 11 and 67 %, again indicating that response depends on disease characteristics of included patients [1, 29–31]. The fact that median overall survival has not been achieved after more than 3 years, that long-term tumor control is possible in rapidly progressing CRPC, and that resolution of skeletal lesions may be observed, indicates a novel therapeutic quality of the present biomodulatory approach in comparison to available therapeutic strategies.

Moreover, encouraging findings of the present trial are a low rate of acute toxicity of the study regimen: the observation of 48 % cumulative grade 3 and 4 toxicities is comparable with those of abiraterone [1]. No drug-related life threatening toxicities have been observed. The tolerability of the regimen is indicated by patient-reported outcomes (quality of life assessments): Early protocol-led dose reductions in response to toxicity – already in case of ≥ grade 2 toxicities - allowed the regimen to be continued over an extended period (>5 years). In contrast to abiraterone which is approved for asymptomatic or less symptomatic patients, the studied approach may rescue patients with rapidly progressive disease (Fig. 5). These findings raise the possibility that this biomodulatory strategy could achieve long-term tumor control with a very low tumor burden irrespectively of the tumor dynamics at baseline.

Previous Phase II trials have shown that glucocorticoid daily or metronomic low-dose cyclophosphamide, or combinations, can achieve a PSA response between 7 and 69 % of patients [16–19, 23, 24]. However, the novel regimen used in the current study may induce an objective response even in patients with rapid PSA doubling times (a majority of patients in the present study population) and extensive metastatic load. In addition, a marked reduction or nearly complete disappearance of bone lesions was observed in bone scans at one center in 6 of 16 patients. Among these patients, two patients showed tumor necrosis with long-term tumor control at a low tumor burden.

This therapeutic schedule included a classic cytotoxic agent at metronomic dose levels, thus avoiding the drug-related toxicities of standard chemotherapy regimens [28, 32]. Although all patients experienced at least one adverse event, drug-related toxicities were generally manageable after prompt dose modifications for events of toxicity. These changes did not appear to markedly limit the efficacy of the regimen. Although 77 % of patients required some type of dose modification or a temporary interruption of study drug, over 60 % of the population showed either a PSA response or maintained a stable disease course. In addition, quality of life was maintained throughout the study and seems to compare with abiraterone [1].

The concerted biomodulatory activity of metronomic low-dose chemotherapy and other biomodulators has been demonstrated previously [8, 21, 24]. Using a similar therapeutic strategy combining etoricoxib, pioglitazone, dexamethasone and metronomically administered capecitabine following first-line chemotherapy, a high PSA response rate was observed (41 %), which was superior to that seen with standard-dose capecitabine alone in historical controls (12 %) [33]. In biomodulatory regimens, the activity of any single drug cannot be defined, since monoactivity is not a prerequisite for concerted activity. Monitoring of biomodulatory activity requires serum analytics of the secretome derived from specific cellular compartments in the tumor, and could provide novel functional signatures [34]. Such an analysis would be necessary to determine which components of the cocktail are redundant or essential, and which have additive or synergistic effects, and may also provide clues for the repurposing of drugs and establishing adaptive trial designs [35, 36].

The central therapeutic problem of tumor heterogeneity, particularly in CRPC [37], may be addressed by targeting selected ‘hallmarks’ of cancer. The rational for targeting single hallmarks of cancer with a biomodulatory therapy approach was driven by the growing knowledge and a better understanding of the biological interrelationships between cancer cells and their environmental stromal and inflammatory cells. Promising clinical data and a favorable toxicity profile indicate that combined modularized therapies need to be explored further, especially for the large and expanding group of elderly and frail patients [38, 39].

## References

[1] Ryan CJ, Smith MR, de Bono JS (2013). Abiraterone in metastatic prostate cancer without previous chemotherapy. N Engl J Med.

[2] Osanto S, Van Poppel H (2012). Emerging novel therapies for advanced prostate cancer. Ther Adv Urol.

[3] Hurwitz M, Petrylak DP (2013). Sequencing of agents for castration-resistant prostate cancer. Oncology (Williston Park).

[4] Reichle A, Vogt T (2008). Systems biology: a therapeutic target for tumor therapy. Cancer Microenviron.

[5] Jain G, Cronauer MV, Schrader M (2012). NF-κB signaling in prostate cancer: a promising therapeutic target?. World J Urol.

[6] Kantoff PW, Higano CS, Shore ND (2010). Sipuleucel-T immunotherapy for castration-resistant prostate cancer. N Engl J Med.

[7] Scher HI, Halabi S, Tannock I (2008). Design and end points of clinical trials for patients with progressive prostate cancer and castrate levels of testosterone: recommendations of the prostate cancer clinical trials working group. J Clin Oncol.

[8] Walter B, Rogenhofer S, Vogelhuber M (2010). Modular therapy approach in metastatic castration-refractory prostate cancer. World J Urol.

[9] Ustach CV, Huang W, Conley-LaComb MK (2010). A novel signaling axis of matriptase/PDGF-D/ß-PDGFR in human prostate cancer. Cancer Res.

[10] Mathew P, Thall PF, Jones D (2004). Platelet-derived growth factor receptor inhibitor imatinib mesylate and docetaxel: a modular phase I trial in androgen-independent prostate cancer. J Clin Oncol.

[11] Kim SJ, Uehara H, Yazici S (2006). Targeting platelet-derived growth factor receptor on endothelial cells of multidrug-resistant prostate cancer. J Natl Cancer Inst.

[12] Nakamura Y, Suzuki T, Sugawara A (2009). Peroxisome proliferator-activated receptor gamma in human prostate carcinoma. Pathol Int.

[13] Matsuyama M, Yoshimura R (2008) Peroxisome proliferator-activated receptor-gamma is a potent target for prevention and treatment in human prostate and testicular cancer. PPAR Res:249849 10.1155/2008/249849 PMC2248699 18317513

[14] Smith MR, Manola J, Kaufman DS (2004). Rosiglitazone versus placebo for men with prostate carcinoma and a rising serum prostate-specific antigen level after radical prostatectomy and/or radiation therapy. Cancer.

[15] Shockley KR, Lazarenko OP, Czernik PJ (2009). PPARgamma2 nuclear receptor controls multiple regulatory pathways of osteoblast differentiation from marrow mesenchymal stem cells. J Cell Biochem.

[16] Storlie JA, Buckner JC, Wiseman GA (1995). Prostate specific antigen levels and clinical response to low dose dexamethasone for hormone-refractory metastatic prostate carcinoma. Cancer.

[17] Nishimura K, Nonomura N, Yasunaga Y (2000). Low doses of oral dexamethasone for hormone-refractory prostate carcinoma. Cancer.

[18] Keith BD (2008). Systematic review of the clinical effect of glucocorticoids on nonhematologic malignancy. BMC Cancer.

[19] Komiya A, Shimbo M, Suzuki H, Imamoto T, Kato T, Fukasawa S, Kamiya N, Naya Y, Mori I, Ichikawa T (2010). Oral low-dose dexamethasone for androgen-independent prostate cancer patients. Oncol Lett.

[20] Khor LY, Bae K, Pollack A (2007). COX-2 expression predicts prostate-cancer outcome: analysis of data from the RTOG 92–02 trial. Lancet Oncol.

[21] Emmenegger U, Chow A, Bocci G, Reichle A (2010). The biomodulatory capacities of low-dose metronomic chemotherapy: complex modulation of the tumor microenvironment. From molecular to modular tumor therapy.

[22] Feyerabend S, Feil G, Krug J (2007). Cytotoxic effects of treosulfan on prostate cancer cell lines. Anticancer Res.

[23] Nelius T, Rinard K, Filleur S (2011). Oral/metronomic cyclophosphamide-based chemotherapy as option for patients with castration-refractory prostate cancer: review of the literature. Cancer Treat Rev.

[24] Glode LM, Barqawi A, Crighton F (2003). Metronomic therapy with cyclophosphamide and dexamethasone for prostate carcinoma. Cancer.

[25] Heidenreich A, Aus G, Bolla M (2008). EAU guidelines on prostate cancer. Eur Urol.

[26] European Organisation for Research and Treatment of Cancer (EORTC) http://www.eortc.be/home/qol/files/SCManualQLQ-C30.pdf Accessed 4 September 2012

[27] Kelly WK, Scher HI, Mazumdar M, Vlamis V, Schwartz M, Fossa SD (1993). Prostate-specific antigen as a measure of disease outcome in metastatic hormone-refractory prostate cancer. J Clin Oncol.

[28] Berthold DR, Pond GR, Soban F (2008). Docetaxel plus prednisone or mitoxantrone plus prednisone for advanced prostate cancer: updated survival in the TAX 327 study. J Clin Oncol.

[29] Ryan CJ, Smith MR, Fong L, Rosenberg JE, Kantoff P, Raynaud F (2010). Phase I clinical trial of the CYP17 inhibitor abiraterone acetate demonstrating clinical activity in patients with castration-resistant prostate cancer who received prior ketoconazole therapy. J Clin Oncol.

[30] Reid AH, Attard G, Danila DC, Oommen NB, Olmos D, Fong PC (2010). Significant and sustained antitumor activity in post-docetaxel, castration-resistant prostate cancer with the CYP17 inhibitor abiraterone acetate. J Clin Oncol.

[31] Sabbatini P, Larson SM, Kremer A, Zhang ZF, Sun M, Yeung H, Imbriaco M, Horak I, Conolly M, Ding C, Ouyang P, Kelly WK, Scher HI (1999). Prognostic significance of extent of disease in bone in patients with androgen-independent prostate cancer. J Clin Oncol.

[32] Petrylak DP, Tangen CM, Hussain MH (2004). Docetaxel and estramustine compared with mitoxantrone and prednisone for advanced refractory prostate cancer. N Engl J Med.

[33] Morant R, Bernhard J, Dietrich D (2004). Capecitabine in hormone-resistant metastatic prostatic carcinoma—a phase II trial. Br J Cancer.

[34] Pitteri SJ, Kelly-Spratt KS, Gurley KE (2011). Tumor microenvironment-derived proteins dominate the plasma proteome response during breast cancer induction and progression. Cancer Res.

[35] Oprea TI, Bauman JE, Bologa CG (2011). Drug repurposing from an academic perspective. Drug Discov Today Ther Strat.

[36] Berry DA (2011). Adaptive clinical trials in oncology. Nat Rev Clin Oncol.

[37] Squire JA, Park PC, Yoshimoto M (2011). Prostate cancer as a model system for genetic diversity in tumors. Adv Cancer Res.

[38] Bellmunt J (2008). Chemotherapy for prostate cancer in senior adults: are we treating the elderly or the frail?. Eur Urol.

[39] Koroukian SM, Murray P, Madigan E (2006). Comorbidity, disability, and geriatric syndromes in elderly cancer patients receiving home health care. J Clin Oncol.

